# Supplementary material for: Memory encoding reprograms neuronal transcriptional responses via durable chromatin remodeling
Source: bioRxiv. 2026 Jul 30:2026.07.29.741555. Preprint. [Version 1] doi: 10.64898/2026.07.29.741555 (PMC13484195; doi:10.64898/2026.07.29.741555)
Supplement: Supplementary file 1 [file NIHPP2026.07.29.741555v1-supplement-1.pdf]

bioRxiv preprint doi: https://doi.org/10.64898/2026.07.29.741555; this version posted July 31, 2026. The copyright holder for this preprint (which was not certified by peer review) is the author/funder, who has granted bioRxiv a license to display the preprint in perpetuity. It is made available under a CC-BY 4.0 International license.

Supplementary Fig. 1

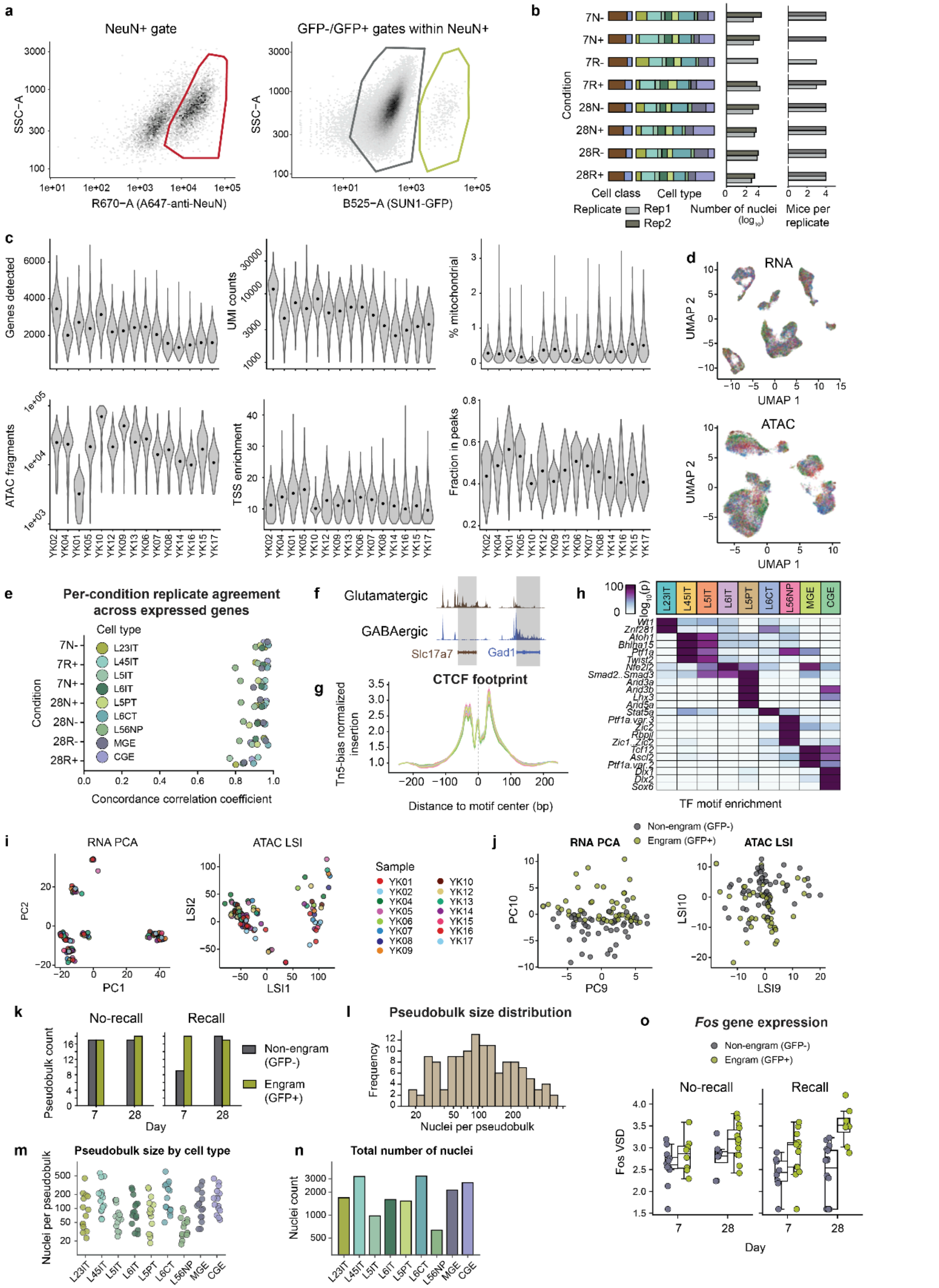

bioRxiv preprint doi: https://doi.org/10.64898/2026.07.29.741555; this version posted July 31, 2026. The copyright holder for this preprint (which was not certified by peer review) is the author/funder, who has granted bioRxiv a license to display the preprint in perpetuity. It is made available under a CC-BY 4.0 International license.

Supplementary Figure 1 | High-quality sorting and sequencing establish a robust mPFC single-nucleus multiome dataset.

a) Representative FANS gating strategy. DAPI+ singlet nuclei were selected after debris and doublet exclusion, followed by isolation of NeuN+ neuronal nuclei and separation into SUN1-GFP− and SUN1-GFP+ gates. Left, side-scatter area (SSC-A; y-axis) versus Alexa Fluor 647-conjugated anti-NeuN fluorescence (R670-A; x-axis), with the NeuN+ gate outlined in red. Right, SSC-A (y-axis) versus SUN1-GFP fluorescence (B525-A; x-axis) within NeuN+ nuclei, with GFP− and GFP+ gates outlined in gray and green, respectively.

b) Mouse, sample, and cell-composition accounting across the eight experimental conditions. Conditions are defined by time after learning (7 or 28 days), recall status (N, no-recall; R, recall), and GFP gate (− or +). Rows indicate condition. The stacked bars show the relative abundance of annotated cell classes and cell types within each condition; colors denote cell identity. Adjacent horizontal bars show the number of nuclei on a $\log_{10}$ scale and the number of mice contributing to each biological replicate. Replicates 1 and 2 are distinguished by dark- and light-gray shading, respectively.

c) Per-nucleus RNA and ATAC quality-control metrics for each sample. Samples are shown on the x-axes. From left to right, top row: number of genes detected per nucleus, RNA unique molecular identifier counts, and percentage of RNA counts assigned to mitochondrial genes; bottom row: number of high-quality ATAC fragments, transcription start-site enrichment, and fraction of ATAC fragments overlapping called peaks. Violin widths represent the density of nuclei at each value, and black points indicate sample medians.

d) RNA and ATAC UMAPs colored by sample. Each point denotes one single nucleus. Color legend is shown in panel **i)**.

e) Agreement of gene-expression profiles between biological replicates. The y-axis indicates experimental condition, and the x-axis shows the concordance correlation coefficient between

bioRxiv preprint doi: https://doi.org/10.64898/2026.07.29.741555; this version posted July 31, 2026. The copyright holder for this preprint (which was not certified by peer review) is the author/funder, who has granted bioRxiv a license to display the preprint in perpetuity. It is made available under a CC-BY 4.0 International license.

replicate pseudobulk RNA profiles for the same condition and cell type. Each point represents one cell type, with colors indicating the annotated cell type.

f) Representative aggregate chromatin-accessibility tracks at canonical neuronal marker loci. Tracks show aggregate ATAC insertion coverage for glutamatergic and GABAergic neurons across genomic position at the *Slc17a7* and *Gad1* loci, respectively; shaded regions highlight cell-class-specific accessibility at marker gene bodies.

g) Aggregate CTCF footprint across cell types. The x-axis indicates distance from the center of CTCF motif occurrences, and the y-axis shows Tn5-bias-normalized insertion frequency. Colored lines represent individual cell types. Depletion of insertions at the motif center, flanked by increased insertion frequency, indicates occupancy of CTCF-bound sites.

h) Cell-type-specific enrichment of transcription-factor motifs in accessible chromatin. Columns indicate cell types, grouped into glutamatergic, GABAergic, and non-neuronal classes, and rows indicate transcription-factor motifs. Color represents motif-enrichment significance as $-\log_{10}(P)$, with darker shading indicating stronger enrichment.

i) First two reduced dimensions for RNA and ATAC encoding cell type differences. Each point denotes one pseudobulk and is colored by sample.

j) The 9th and 10th reduced dimensions for RNA and ATAC encode condition-related cell states. Each point denotes one pseudobulk and is colored by engram (green) or non-engram (gray).

k) Summary of pseudobulks retained for downstream analyses after requiring more than 15 nuclei per sample-by-cell-type pseudobulk. The x-axes indicate day after learning and the y-axes indicate the number of retained pseudobulks for no-recall and recall conditions, respectively; gray and green bars denote GFP− and GFP+ populations.

l) Distribution of pseudobulk sizes, with nuclei per pseudobulk on the x-axis and pseudobulk frequency on the y-axis.

bioRxiv preprint doi: https://doi.org/10.64898/2026.07.29.741555; this version posted July 31, 2026. The copyright holder for this preprint (which was not certified by peer review) is the author/funder, who has granted bioRxiv a license to display the preprint in perpetuity. It is made available under a CC-BY 4.0 International license.

m) Pseudobulk sizes per cell type. X-axis, cell type; y-axis, number of nuclei per pseudobulk. Colors indicate cell types as in **g)**.

n) Total number of nuclei per cell type. X-axis, cell type; y-axis, total number of nuclei. Colors indicate cell types as in **g)**.

o) *Fos* is expressed at higher levels in engram than non-engram cells especially at 28 d recall. Y-axes, DESeq2-normalized Fos expression level (VSD). Each point denotes one pseudobulk.

bioRxiv preprint doi: https://doi.org/10.64898/2026.07.29.741555; this version posted July 31, 2026. The copyright holder for this preprint (which was not certified by peer review) is the author/funder, who has granted bioRxiv a license to display the preprint in perpetuity. It is made available under a CC-BY 4.0 International license.

Supplementary Fig. 2

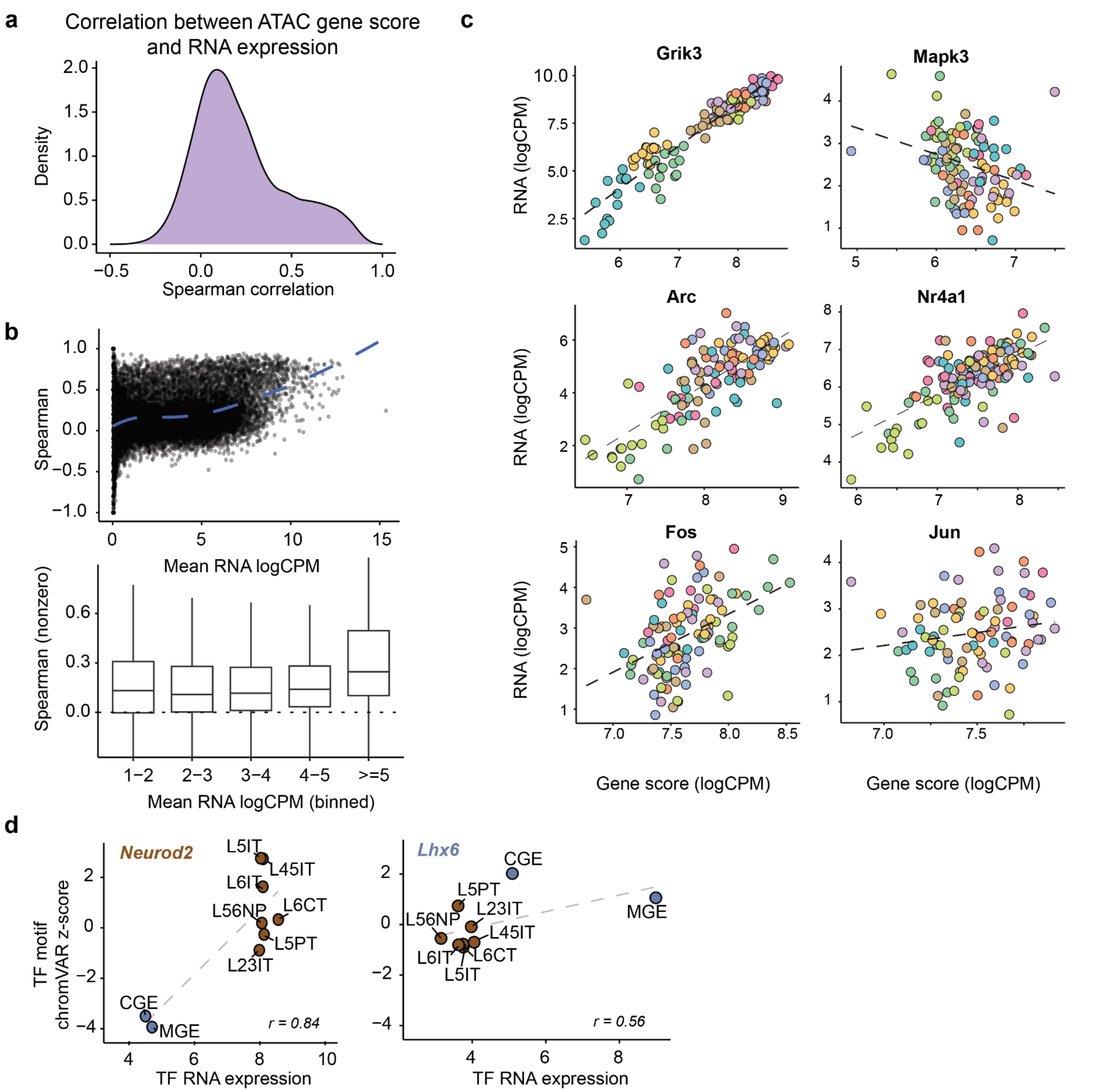

Supplementary Figure 2 | Cross-modality coupling between RNA expression and chromatin accessibility is highly gene dependent.

a) Distribution of Spearman correlations between matched ATAC gene scores and RNA expression across genes. Most genes show modest correlation, while a subset of genes show positive cross-modality coupling.

bioRxiv preprint doi: https://doi.org/10.64898/2026.07.29.741555; this version posted July 31, 2026. The copyright holder for this preprint (which was not certified by peer review) is the author/funder, who has granted bioRxiv a license to display the preprint in perpetuity. It is made available under a CC-BY 4.0 International license.

b) Gene-level RNA–ATAC coupling only modestly increases with expression levels. Top, Spearman correlation between gene score and RNA expression plotted against mean RNA abundance. Bottom, genes binned by mean RNA expression show only slightly stronger modality concordance among more highly expressed genes.

c) Representative genes illustrating diverse RNA–ATAC relationships across nuclei. Canonical cell type markers (e.g. *Grik3*) show tight coupling, and housekeeping genes (e.g. *Mapk3*) show no or negative coupling. Immediate early genes (e.g. *Arc*, *Nr4a1*, *Fos*, and *Jun*) span a spectrum of high to low cross-modality coupling. *Grik3* and *Mapk3* were selected as the highest coupled and lowest coupled genes; *Arc*, *Nr4a1*, *Fos*, and *Jun* were selected as representative immediate early genes. Each dot represents one pseudobulk and is colored by cell type.

d) TF activity inferred from chromVAR deviation scores is correlated with TF RNA expression across cell types for representative lineage-associated transcription factors, including *Neurod2* and *Lhx6*. Each point represents a cell type; labels indicate major neuronal subclasses.

bioRxiv preprint doi: https://doi.org/10.64898/2026.07.29.741555; this version posted July 31, 2026. The copyright holder for this preprint (which was not certified by peer review) is the author/funder, who has granted bioRxiv a license to display the preprint in perpetuity. It is made available under a CC-BY 4.0 International license.

Supplementary Fig. 3

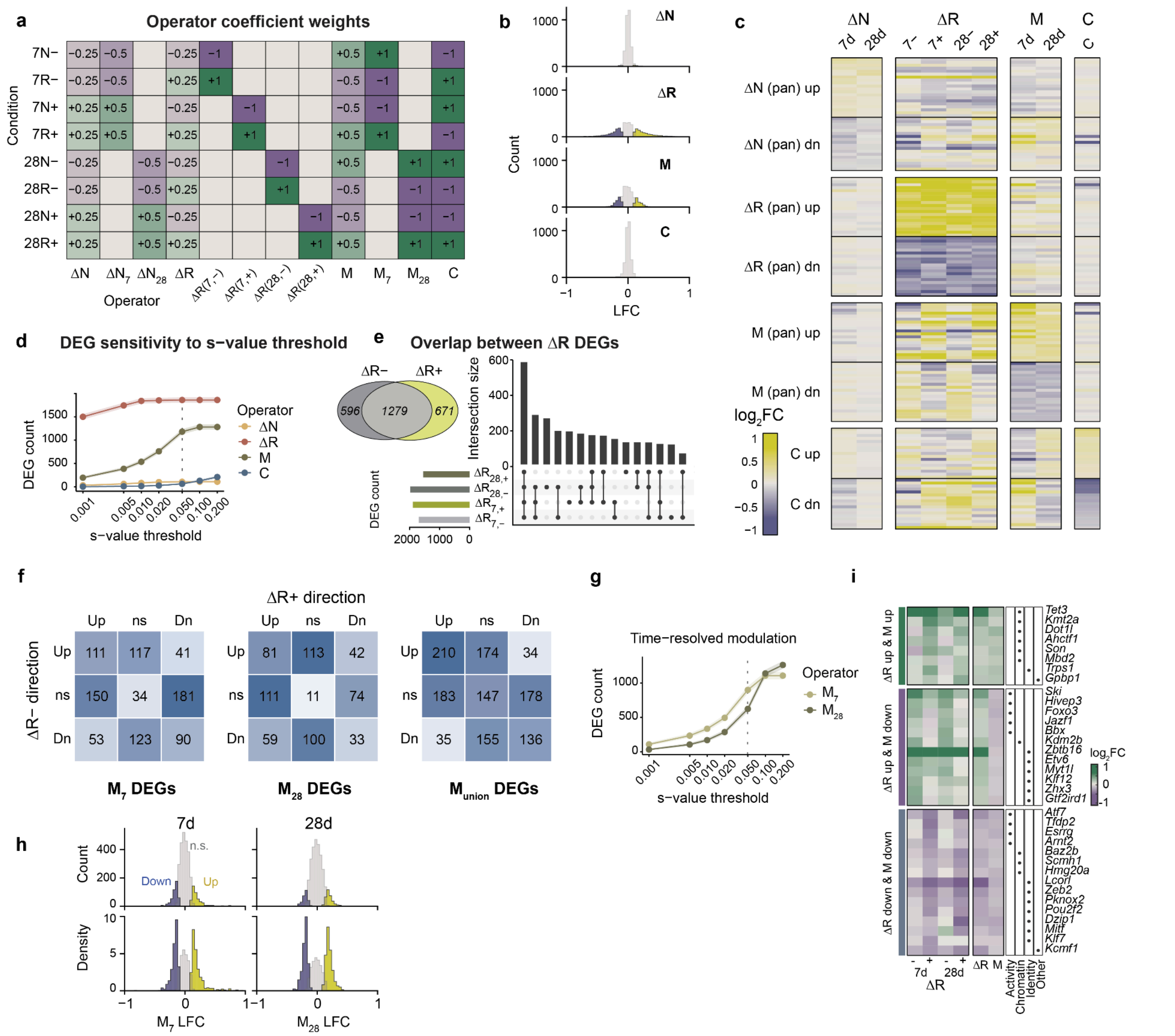

Supplementary Figure 3 | Operator-based differential expression reveals bidirectional engram modulation of recall responses.

a) Linear contrast weights used to estimate coefficients from the eight experimental conditions (rows), including no-recall engram differences at 7 d and 28 d after learning (ΔN_7, ΔN_{28}), recall responses in each time point and gating condition (ΔR), time-resolved engram modulation of recall responses (M_7, M_{28}), their aggregation over time points (M), and the maturation of modulation across consolidation time (C).

bioRxiv preprint doi: https://doi.org/10.64898/2026.07.29.741555; this version posted July 31, 2026. The copyright holder for this preprint (which was not certified by peer review) is the author/funder, who has granted bioRxiv a license to display the preprint in perpetuity. It is made available under a CC-BY 4.0 International license.

b) Distributions of log2 fold-change estimates (x-axis) for *ΔN, ΔR, M*, and *C*, with significant positive and negative effects highlighted. Y-axis, gene counts. Shaded ribbons indicate 95% Wilson confidence intervals for DEG counts. Yellow, upregulated; purple, downregulated; gray, non-significant.

c) Heatmaps of log2 fold changes for genes grouped by significant up- or down-regulation for each regulatory quantity, showing how no-recall engram state, recall response, engram modulation, and maturation effects relate across time points.

d) Sensitivity of DEG counts (y-axis) to s-value threshold (x-axis) for each regulatory quantity (color). Shaded ribbons indicate 95% Wilson confidence intervals for DEG counts.

e) Overlap among recall-response DEGs across time point and gating (engram) conditions, with total *ΔR*+ (recall response in engram cells) and *ΔR*− (recall response in non-engram cells) DEG counts summarized by Venn diagram.

f) Relationship between underlying recall-response directions in engram and non-engram cells for engram modulation DEGs. Heatmaps show genes significant for M_7, M_{28}, or their aggregation over time points (*M*). Rows indicate the direction of the recall response in non-engram cells (*ΔR*−), and columns indicate the direction of the recall response in engram cells (*ΔR*+). “Up” and “Dn” indicate significant recall-induced up- or down-regulation, and “ns” indicates no significant recall response. Numbers indicate the number of modulation DEGs in each *ΔR*−/*ΔR*+ category. Diagonal entries represent concordant recall responses between engram and non-engram cells, whereas off-diagonal entries represent gain, loss, attenuation, potentiation, or reversal of recall responses in engram cells. This classification reveals whether engram modulation reflects enhanced induction, reduced induction, selective induction, enhanced repression, reduced repression, or polarity switching.

g) Sensitivity of time-resolved modulation DEG counts (y-axis) to s-value threshold (x-axis) for M_7 and M_{28}.

bioRxiv preprint doi: https://doi.org/10.64898/2026.07.29.741555; this version posted July 31, 2026. The copyright holder for this preprint (which was not certified by peer review) is the author/funder, who has granted bioRxiv a license to display the preprint in perpetuity. It is made available under a CC-BY 4.0 International license.

h) Distribution of engram-modulation log2 fold changes (LFC) at 7 d and 28 d after learning. Histograms show the full distribution of M_7 and M_{28} LFC across genes. Top row shows gene counts, and bottom row shows the same distributions normalized to density to better compare the shape of significant and non-significant effects. Yellow bars indicate genes with significant positive engram modulation, where recall-induced expression is higher in engram cells than in non-engram cells. Purple bars indicate genes with significant negative engram modulation. Gray bars indicate genes without significant modulation. Vertical lines mark the log2 fold-change positions of significant up- and down-modulated genes. Both time points show bidirectional modulation, with positive and negative interaction effects rather than a single global shift in recall responsiveness.

i) TF gene expression patterns associated with representative regulatory categories. Heatmaps show log2 fold changes for transcription factor genes grouped by their differential expression patterns across recall response and engram modulation quantities. Rows are TF genes, and columns show recall-response coefficients at 7 d and 28 d in non-engram and engram cells ($\Delta R-$ and $\Delta R+$), followed by engram modulation (M). Green indicates increased TF expression and purple indicates decreased TF expression. TFs are grouped into three illustrative patterns: genes with coordinated recall induction and positive modulation (ΔR up & M up), genes with recall induction but negative modulation (ΔR up & M down), and genes with recall repression and negative modulation (ΔR down & M down). The annotation bar on the right summarizes broad functional classes for each TF, including activity-associated, chromatin-related, identity-related, and other regulatory categories. Filled dots indicate membership in each functional annotation class.

bioRxiv preprint doi: https://doi.org/10.64898/2026.07.29.741555; this version posted July 31, 2026. The copyright holder for this preprint (which was not certified by peer review) is the author/funder, who has granted bioRxiv a license to display the preprint in perpetuity. It is made available under a CC-BY 4.0 International license.

Supplementary Fig. 4

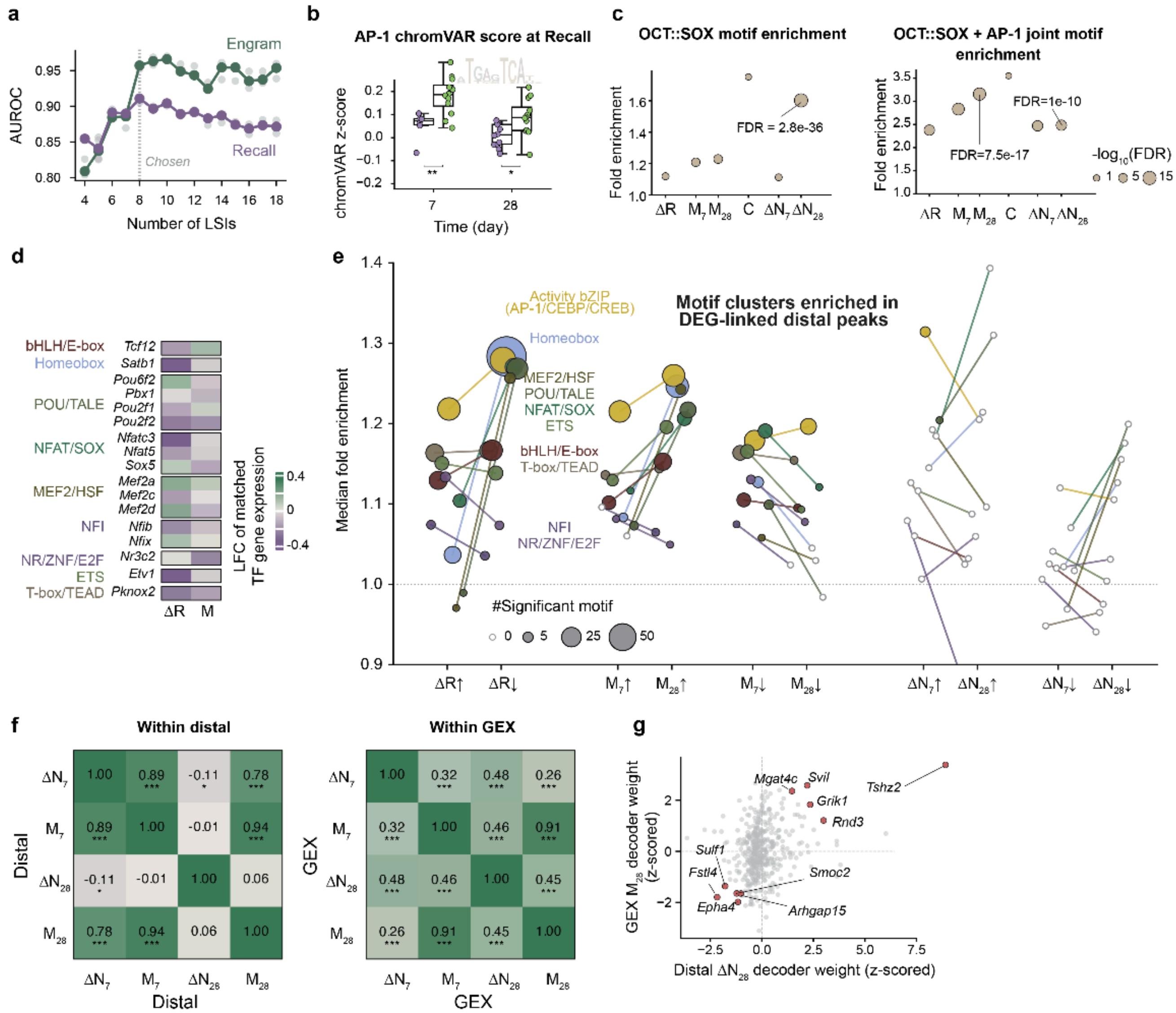

Supplementary Figure 4 | Decoder analysis links persistent chromatin engram signatures to motif programs and transcriptional modulation.

a) AUROC as a function of the number of distal LSI dimensions included in the decoder grid search. Performance is shown as the median across five cross-validation folds for the engram decoder (green) and recall decoder (purple); non-median fold values are shown as gray dots. The selected number of LSI dimensions is indicated by the gray vertical dashed line.

bioRxiv preprint doi: https://doi.org/10.64898/2026.07.29.741555; this version posted July 31, 2026. The copyright holder for this preprint (which was not certified by peer review) is the author/funder, who has granted bioRxiv a license to display the preprint in perpetuity. It is made available under a CC-BY 4.0 International license.

b) AP-1 motif activity at recall, measured by chromVAR z-score, stratified by time point and distal engram decoder score. Boxplots show AP-1 chromVAR scores in nuclei grouped by decoder-predicted chromatin engram state, showing higher AP-1 accessibility in engram cells compared to non-engram cells at 7 d and 28 d.

c) The canonical OCT::SOX motif is enriched in no-recall engram at 28 d, and the OCT::SOX–AP-1 joint motif is enriched in both recall-time modulation and no-recall engram. Motif enrichment for OCT::SOX and OCT::SOX–AP-1 joint motifs across distal peak sets linked to ΔN, ΔR, and M. Dot position on y-axis indicates fold enrichment, dot size indicates −log10(FDR), and labels highlight significant enrichment in persistent chromatin engram signatures, especially ΔN_{28}. The OCT::SOX–AP-1 joint motif was defined as a given distal peak having both OCT::SOX and AP-1 motifs.

d) TF-encoding genes with concordant evidence from motif enrichment and gene expression. Heatmap shows log2 fold changes for TF genes in ΔR and M expression estimates, with selected motif families labeled on the left.

e) Motif cluster enrichment across DEG-linked distal peak sets separated by regulatory quantity and direction. Each point represents a motif cluster, y-axis indicates median enrichment, point size indicates the number of significant motifs in the cluster, and lines connect related motif clusters across regulatory quantities. Motif families enriched in recall response, engram modulation, and no-recall engram chromatin signatures include activity bZIP/AP-1, homeobox, POU/TALE, MEF2/HSF, FOX/SOX, ETS, bHLH/E-box, NFI, NR/ZNF, and T-box/TEAD.

f) Distal ΔN_{28} is distinct from the other distal chromatin signatures and is slightly anti-correlated with the early distal engram signature (ΔN_7), whereas gene expression signatures show broader positive coupling across contrasts. Heatmaps show pairwise correlations between decoder weight vectors for ΔN_7, M_7, ΔN_{28}, and M_{28} within distal chromatin features (left) and within gene expression features (right). Each row and column represents a quantity-specific decoder weight vector, and each cell shows the correlation between the corresponding pair of vectors. In distal chromatin, ΔN_7 is strongly

bioRxiv preprint doi: https://doi.org/10.64898/2026.07.29.741555; this version posted July 31, 2026. The copyright holder for this preprint (which was not certified by peer review) is the author/funder, who has granted bioRxiv a license to display the preprint in perpetuity. It is made available under a CC-BY 4.0 International license.

aligned with M_7 and M_{28}, and M_7 is strongly aligned with M_{28}, indicating that early engram-associated distal accessibility resembles both early and late modulation-associated distal programs. By contrast, distal ΔN_{28} separates from this shared axis, showing weak or negative correlation with ΔN_7 and M_7 and little correlation with M_{28}. In gene expression, all quantity-specific signatures are positively correlated, with especially strong similarity between M_7 and M_{28}, indicating that transcriptional weight directions are less separated than distal chromatin weight directions. * $p < 0.05$; ** $p < 0.01$; *** $p < 0.001$ for a null of no correlation.

g) Relationship between distal chromatin ΔN_{28} decoder weights and gene expression M_{28} decoder weights without correcting for gene expression ΔN_{28} decoder weights. Each point represents a gene or linked feature, with selected genes labeled. The scatter compares persistent distal chromatin engram signatures with later transcriptional modulation. Top 5 genes in concordance are highlighted in red and labeled.

bioRxiv preprint doi: https://doi.org/10.64898/2026.07.29.741555; this version posted July 31, 2026. The copyright holder for this preprint (which was not certified by peer review) is the author/funder, who has granted bioRxiv a license to display the preprint in perpetuity. It is made available under a CC-BY 4.0 International license.

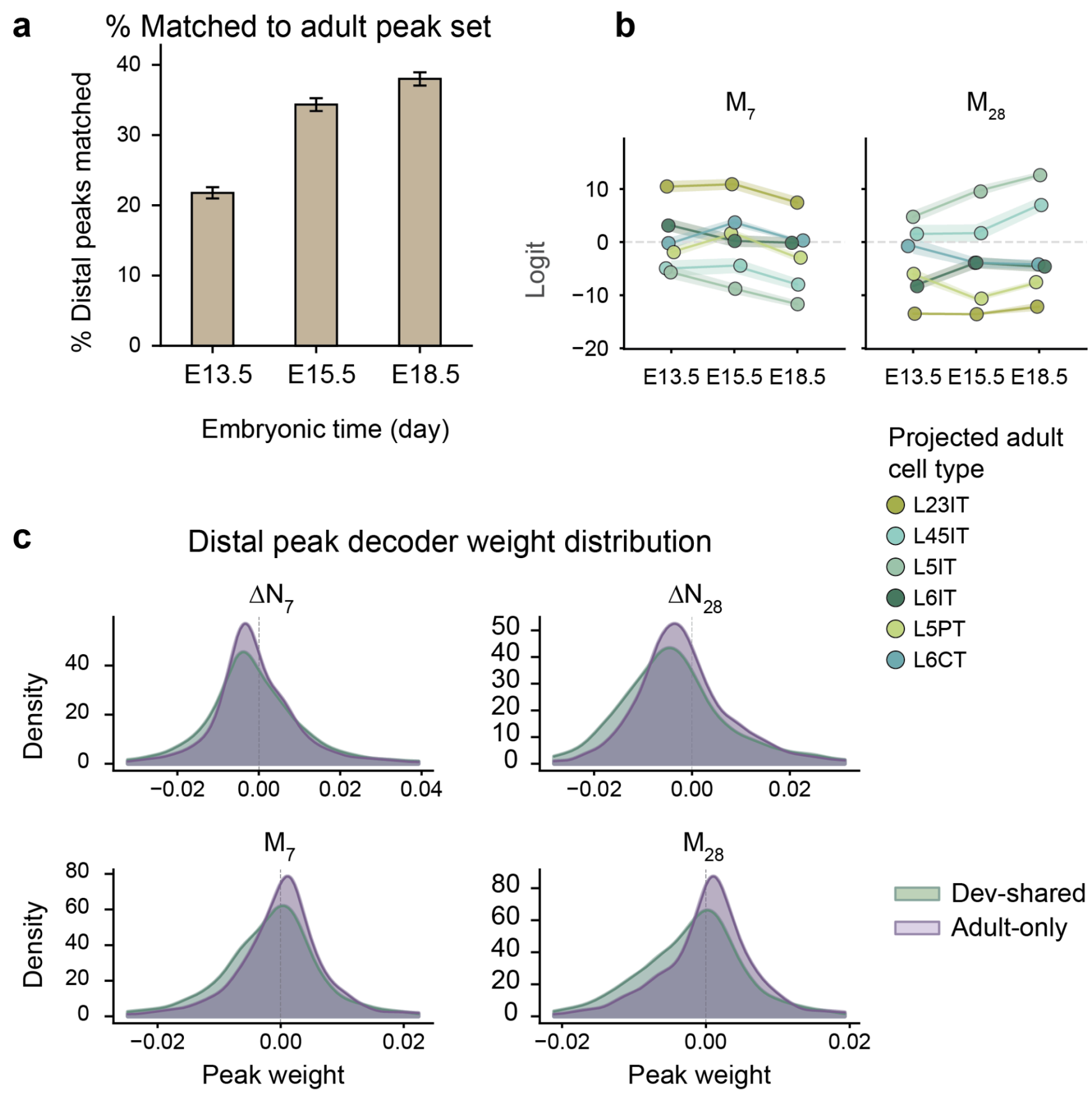

Supplementary Figure 5 | Adult engram and modulation chromatin programs partially reuse embryonic regulatory elements.

a) Fraction of adult distal peaks matched to embryonic developmental peak sets increases from E13.5, E15.5, to E18.5. Bars show the percentage of adult distal peaks that overlap accessible chromatin regions detected at each embryonic time point, indicating that a subset of adult regulatory elements used in engram and modulation analyses is shared with developmental chromatin programs. Error bars represent 95% Wilson confidence intervals.

b) Projection of M_7 and M_{28} distal decoder weights onto embryonic developmental time points. Lines connect projected cell types across developmental stages, showing whether peaks associated with engram modulation in adult mPFC align with temporally changing embryonic accessibility programs.

bioRxiv preprint doi: https://doi.org/10.64898/2026.07.29.741555; this version posted July 31, 2026. The copyright holder for this preprint (which was not certified by peer review) is the author/funder, who has granted bioRxiv a license to display the preprint in perpetuity. It is made available under a CC-BY 4.0 International license.

Colors represent projected cell types as in **Fig. 4b**. Shaded ribbons represent standard errors over cross-validation folds.

c) Distribution of distal peak decoder weights for development-shared and adult-only peaks across ΔN_7, ΔN_{28}, M_7, and M_{28}. Density plots compare peak weights for distal elements that are shared with embryonic developmental peak sets versus peaks detected only in the adult dataset.

bioRxiv preprint doi: https://doi.org/10.64898/2026.07.29.741555; this version posted July 31, 2026. The copyright holder for this preprint (which was not certified by peer review) is the author/funder, who has granted bioRxiv a license to display the preprint in perpetuity. It is made available under a CC-BY 4.0 International license.

Supplementary Fig. 6

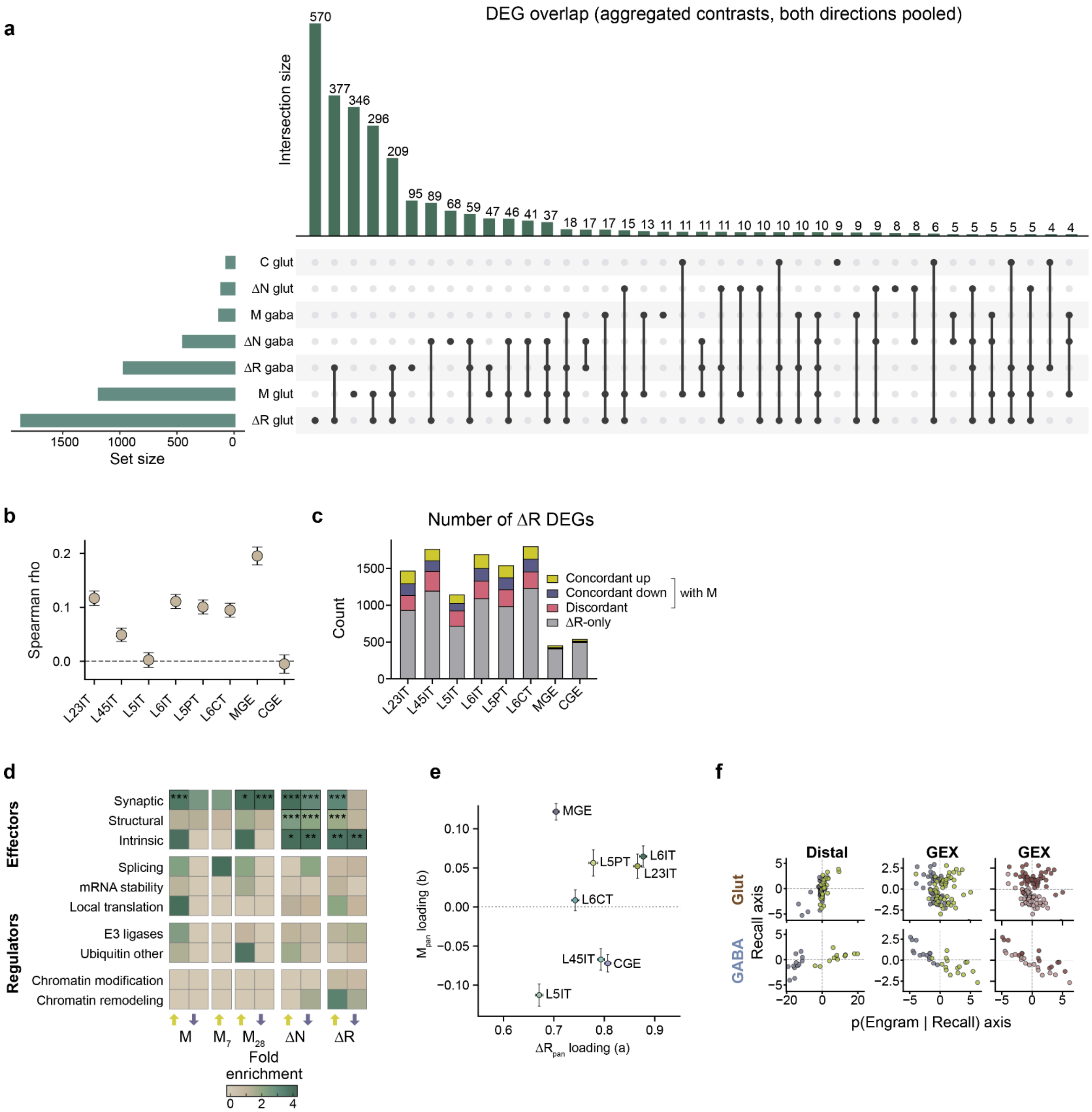

Supplementary Figure 6 | Engram modulation is weaker in GABAergic neurons despite shared recall-response programs.

a) Overlap of DEGs across glutamatergic and GABAergic regulatory quantities. UpSet plot shows intersections among *ΔR, M, ΔN*, and *C* DEG sets for glutamatergic and GABAergic neurons, with directions pooled across up- and down-regulated genes. Horizontal bars show total set sizes, vertical

bioRxiv preprint doi: https://doi.org/10.64898/2026.07.29.741555; this version posted July 31, 2026. The copyright holder for this preprint (which was not certified by peer review) is the author/funder, who has granted bioRxiv a license to display the preprint in perpetuity. It is made available under a CC-BY 4.0 International license.

bars show intersection sizes, and connected dots indicate the DEG sets contributing to each intersection.

b) Correlation between ΔR and M gene expression log2 fold changes across matched cell types. Each point represents a neuronal cell type, and the y-axis shows Spearman's rho between gene expression LFC effect sizes of aggregated ΔR and M, derived from the DESeq2 model as in **Fig. 2**. Error bars indicate the standard error of Spearman's rho, estimated via Monte Carlo simulation (n=1000) by sampling from each gene's $\log_2$ fold change distribution using its shrinkage-derived standard error.

c) Number of recall-response DEGs across neuronal cell types. Stacked bars show genes significant for ΔR in each cell type, separated into genes concordant with the pan-cell-class M direction, discordant with the M direction, or significant only in ΔR but not M. Glutamatergic cell types were compared to pan-glutamatergic M, and GABAergic to pan-GABAergic M.

d) Functional enrichment of DEGs in GABAergic neurons. Heatmap shows fold enrichment for curated effector and regulator categories across M, M_7, M_{28}, ΔN, and ΔR. Colormap, fold enrichment. * FDR < 0.05; ** FDR < 0.01; *** FDR < 0.001.

e) Relationship between loadings in pan-cell-class recall-response and engram-modulation for cell-type-specific recall-response LFC vectors. Points represent neuronal cell types, with x-axis showing ΔR_{pan} loading and y-axis showing M_{pan} loading in a linear model of $\Delta R_{cell\ type} \sim \Delta R_{pan} + M_{pan}$. Error bars indicate uncertainty of model estimates.

f) Projection of glutamatergic and GABAergic cells onto decoder axes. Scatter plots show cells projected onto the logit corresponding to *p(Engram | Recall)* and an orthogonal recall-associated axis for distal chromatin and gene expression features. Panels compare glutamatergic and GABAergic distributions, illustrating that engram-related structure is more separable in GABAergic neurons compared to glutamatergic neurons, and that both cell classes share a common engram-related dimension in the high-dimensional distal chromatin and gene expression space.

bioRxiv preprint doi: https://doi.org/10.64898/2026.07.29.741555; this version posted July 31, 2026. The copyright holder for this preprint (which was not certified by peer review) is the author/funder, who has granted bioRxiv a license to display the preprint in perpetuity. It is made available under a CC-BY 4.0 International license.

Supplementary Tables

Supplementary Table 1 | Sample metadata

Library ID	Condition	Mouse pool ID	Age (week)	Male	Female	Total	Used for DEG analysis	Used for decoder
YK01	7N+	B2	13	4	0	4	No	Yes
YK02	7N-	B4	13	0	4	4	Yes	Yes
YK03	7N+	B4	13	0	4	4	Yes	Yes
YK04	7N-	B10	20	2	2	4	Yes	Yes
YK05	7N+	B10	20	2	2	4	Yes	Yes
YK06	7R-	B12	15	1	2	3	Yes	Yes
YK07	7R+	B12	15	1	2	3	Yes	Yes
YK08	7R+	B14	13	2	2	4	Yes	Yes
YK09	28N+	B3	16	0	4	4	Yes	Yes
YK10	28N-	B7	19	2	2	4	No	Yes
YK11	28N+	B7	19	2	2	4	Yes	Yes
YK12	28N-	B11	18	2	2	4	Yes	Yes
YK13	28N+	B11	18	2	2	4	Yes	Yes
YK14	28R-	B13	15	2	2	4	Yes	Yes
YK15	28R+	B13	15	2	2	4	Yes	Yes
YK16	28R-	B15	12	2	2	4	Yes	Yes
YK17	28R+	B15	12	2	2	4	Yes	Yes

bioRxiv preprint doi: https://doi.org/10.64898/2026.07.29.741555; this version posted July 31, 2026. The copyright holder for this preprint (which was not certified by peer review) is the author/funder, who has granted bioRxiv a license to display the preprint in perpetuity. It is made available under a CC-BY 4.0 International license.

Supplementary Table 2 | Decoder coefficient matrix

	ΔR	ΔN_{mean}	ΔN_{time}	M_{mean}	M_{time}
7N-	0	0	0	0	0
7R-	1	0	0	0	0
7N+	0	1	-1	0	0
7R+	1	1	-1	1	-1
28N-	0	0	0	0	0
28R-	1	0	0	0	0
28N+	0	1	1	0	0
28R+	1	1	1	1	1
